# Dialog beyond the Grave: Necrosis in the Tumor Microenvironment and Its Contribution to Tumor Growth

**DOI:** 10.3390/ijms24065278

**Published:** 2023-03-09

**Authors:** Emilija Zapletal, Tea Vasiljevic, Pierre Busson, Tanja Matijevic Glavan

**Affiliations:** 1 Laboratory for Personalized Medicine, Division of Molecular Medicine, Rudjer Boskovic Institute, Bijenicka 54, 10000 Zagreb, Croatia; 2 CNRS UMR 9018-METSY, Université Paris-Saclay, Gustave Roussy, 39 Rue Camille Desmoulins, F-94805 Villejuif, France

**Keywords:** necrosis, damage-associated molecular patterns (DAMPs), Toll-like receptors, cancer, tumor microenvironment (TME), HMGB1, RAGE

## Abstract

Damage-associated molecular patterns (DAMPs) are endogenous molecules released from the necrotic cells dying after exposure to various stressors. After binding to their receptors, they can stimulate various signaling pathways in target cells. DAMPs are especially abundant in the microenvironment of malignant tumors and are suspected to influence the behavior of malignant and stromal cells in multiple ways often resulting in promotion of cell proliferation, migration, invasion, and metastasis, as well as increased immune evasion. This review will start with a reminder of the main features of cell necrosis, which will be compared to other forms of cell death. Then we will summarize the various methods used to assess tumor necrosis in clinical practice including medical imaging, histopathological examination, and/or biological assays. We will also consider the importance of necrosis as a prognostic factor. Then the focus will be on the DAMPs and their role in the tumor microenvironment (TME). We will address not only their interactions with the malignant cells, frequently leading to cancer progression, but also with the immune cells and their contribution to immunosuppression. Finally, we will emphasize the role of DAMPs released by necrotic cells in the activation of Toll-like receptors (TLRs) and the possible contributions of TLRs to tumor development. This last point is very important for the future of cancer therapeutics since there are attempts to use TLR artificial ligands for cancer therapeutics.

## 1. Introduction

For a long time, biological investigations of malignant diseases have been mainly focused on the malignant cells and their genetic and epigenetic alterations. Since the first years of the 2000s, more emphasis has been placed on the “dialog” between malignant cells and stromal cells. In this review, we will try to illustrate this crosstalk between necrotic cells and live cells (malignant and stromal), and its consequences for tumor growth.

Cell death by necrosis can be caused by acute physical (extreme temperature, radiation, electric shock) or chemical injuries, mechanical trauma, infections, toxins, and ischemia [1]. Necrosis is characterized by cell and organelle swelling, nuclear condensation (pyknosis), and loss of plasma membrane integrity. Swollen cells and membrane rupture caused by membrane permeabilization represent an early event in necrotic cells, while the same event in apoptotic cells occurs later [2,3]. Necrosis is a quasi-constant phenomenon in solid tumors, although of variable magnitude. Sometimes it is amplified by anti-tumor therapy. One consistent characteristic of necrosis is the fact that it simultaneously affects a large number of cells, whereas apoptosis generally affects scattered, individual cells [4]. As soon as a solid tumor reaches 4 mm in diameter, the core region of the tumor, due to inadequate vascularization, experiences hypoxia and nutrient deprivation that leads to necrosis [5]. The presence of hypoxia and necrosis is one key difference between tumors and normal tissues that could potentially become an angle of attack for more selective cancer therapies. Currently, tumor hypoxia is mainly a factor of therapeutic resistance. Hypoxic cells are 2- to 3-fold more resistant than normoxic cells to radiotherapy due to the lack of oxygen in their environment [6]. Tumor core hypoxic cells also exhibit increased chemoresistance because less of the drug reaches the core due to poor vascularization and high interstitial fluid pressures [7]. New strategies to selectively target those resistant hypoxic tumor cells are cytotoxic drugs that only work under hypoxic conditions, gene therapy constructs with hypoxia-inducible promotors, and obligate anaerobic bacteria delivery vectors that selectively colonize and replicate within the tumor necrotic core [6,7,8].

Efficient anti-tumor therapy sometimes leads to necrotic cell death, which unlike apoptosis, induces inflammatory responses that may contribute to tumor regression [4]. However, spontaneous necrosis is often correlated with rapid tumor growth [4,5,9]. One explanation is the fact that angiogenesis is often all the more insufficient and unsuitable as the malignant cells proliferate rapidly. On the other hand, necrosis by itself, in a kind of vicious circle, may promote malignant cell proliferation, genomic instability, metastasis and even (imperfect) angiogenesis. There is a close link between the undesirable effects of necrosis and those of chronic inflammation [4,5,9].

## 2. Tumor Necrosis in Human Solid Tumors: Clinical and Pathological Aspects

Necrosis is an important prognostic factor. Richards et al. reported in 2011 that there are over 50 published studies confirming the prognostic value of tumor necrosis in patients with solid organ malignant diseases [10]. Nowadays, its number has increased as other meta-analyses and other studies have emerged [11,12,13,14].

### 2.1. Necrosis Assessment by Medical Imaging 

#### 2.1.1. Conventional Magnetic Resonance (MR) Imaging

Conventional contrast enhanced MR imaging has been the main technique to diagnose tumor and its recurrence [15]. Necrosis at initial MRI is often associated with metastatic disease at presentation or disease progression and is mostly studied in renal cell carcinoma and glioblastoma. Increase of necrosis, or de novo necrosis development during treatment, is a negative prognostic factor for some tumors and it precedes progression. Detection of changes (of necrosis) on MRI scans that precede tumor progression are clinically important because they save time and give the opportunity to change ineffective treatment [16,17]. However, MRI offers only limited power to differentiate between tumor recurrence and necrosis because they manifest similar features on MRI scans [18].

#### 2.1.2. Functional Imaging Techniques

Advanced imaging techniques that are available for discrimination between treatment outcomes include functional magnetic resonance (MR) perfusion techniques, diffusion-weighted imaging (DWI), magnetic resonance spectroscopy (MRS), positron emission tomography (PET), and single photon emission CT (SPECT) [18,19]. PET and SPECT use radiopharmaceuticals to image the different functional properties of organs and tissues. Compared with necrotic regions, tumor recurrence is characterized by an increased metabolism of the growing tumor, which is expected to result in higher tracer uptake. A commonly used tracer for PET is fludeoxyglucose (18F-FDG), a glucose analogue, while the potential of amino acid analogs has also been explored [18]. Multimodal functional imaging can give increased accuracy when structural MRI or PET are combined with perfusion techniques or MRS [18,19]. However, the use of multiple techniques is costly, time-consuming, limited by low accessibility, and remains impractical in the clinical setting [18].

#### 2.1.3. Molecular Imaging Techniques

There are several classes of necrosis-avid contrast agents that can bind cell components exposed by the loss of membrane integrity, and can thus be used to visualize necrosis [20,21]. Rhein and its derivatives are anthraquinone compounds, a class of DNA intercalators that exhibit intrinsic fluorescence [22]. They are used for non-invasive visualization of myocardial necrosis [23] and necrosis in tumors [24,25], therefore, representing a base for the development new PET and SPECT tracers [20,26].

A promising PET tracer for in vivo detection of tumor necrosis is gallium-68-labeled IRDye800CW, a cyanine-based fluorescent dye that exhibits excellent necrosis avidity by binding cytoplasmic proteins [21]. Another cyanine dye that shows avidity for necrotic tissues is an FDA-approved fluorescent probe indocyanine green (ICG). It can selectively bind necrotic tissue due to interactions with lipoproteins and phospholipids. In the preclinical model of fluorescence molecular imaging, ICG was used in the hybrid modality system PET/CT/FMI for imaging tumor progression and therapy outcomes in vivo [27]. 

The known in situ biomarkers of necrosis are also exploited to visualize necrosis or to target therapy to the necrotic area of solid tumors. In situ biomarkers of necrosis, targeted with antibodies and different small molecular compounds, include DNA/histone H1 complex, exposed DNA, heat shock protein 90 (HSP90), fumarase, and high mobility group box1 (HMGB1). Several molecular imaging probes designed for imaging and/or delivery of therapeutics are assessed in preclinical and clinical trials (for an extensive review see [20]).

### 2.2. Necrosis Assessment by Histopathological Examination 

A typical feature of malignant tumors is the formation of large necrotic areas, mainly due to inadequate clearance by the macrophage phagocytic response in addition to hypoxia. In histological sections, tumor necrosis has two morphologically distinct patterns. Coagulative necrosis is characterized by clusters of eosinophilic and anucleated necrotic cells while the architecture of tissue is still preserved. This necrosis is typical for ischemia-induced injuries in all organs except the brain [1,28]. In the brain, hypoxic cell death often causes liquefactive necrosis in which necrotic tissue is completely degraded into a liquid viscous mass, similar to that seen in bacterial infections [1,29].

### 2.3. Circulating Biomarkers—Biological Assays for Necrosis Assessment 

Assays used for necrosis measurement are based on the loss of membrane integrity. Accidental necrosis is characterized by a sudden release of cell content that finds its way into the blood flow. Circulating biomarkers are a measure of excessive cell death and inadequate phagocytic clearance [2]. The most commonly used in vitro assay for assessing the necrosis amounts is the release of cytosolic enzyme lactate dehydrogenase (LDH) [30]. LDH is present in almost all cells, and released molecules are detectable in peripheral blood. Elevated plasma LDH is a sign of necrosis and tissue damage. Free HMGB1 is passively released from necrotic or damaged cells. It is not released from apoptotic cells even after the secondary necrosis due to its binding to nucleosomes [31]. Free circulating HMGB1 can be measured by ELISA [2]. Cytokeratin 18 (CK18) is a structural protein of epithelial cells, highly expressed in many epithelial tissues, including the liver, intestine, lung, kidney, and endocrine glands, as well as many solid tumors [2,32]. Necrotic cells release an unmodified form of CK18, while apoptotic cells release a caspase-cleaved form, whose epitope can be detected with M30 ELISA. M65 ELISA quantitates total circulating CK18 i.e., caspase-cleaved and non-cleaved form [33]. Using both ELISAs, it is possible to estimate the release of non-cleaved CK18, which provides an estimation of necrosis occurring in distant foci [2]. Tumor-derived cell-free DNAs (cfDNAs) are of increasing interest for the early detection of various tumors and their metastasis. cfDNAs found in serum consist mainly of ~166 bp long fragments that correspond to the length of DNA wrapped around nucleosomes. Those fragments are assumed to be released by apoptosis and may contain tumor-specific sequences [2,34]. Longer and shorter fragments were detected in several studies and ascribed to necrosis [35]. A study using massively parallel sequencing proposed that shifts in cfDNA fragment sizes can be used for disease follow-up in hepatocellular carcinoma patients [36]. In some tumors, necrosis may be the main mechanism contributing to cfDNA release in response to ionizing radiation [35]. Another form of nucleic acids available to assay from peripheral blood is extracellular microRNAs (ex-miRNA), which are ribonucleoprotein particles containing short (~20 nucleotides long) non-coding RNA molecules that regulate gene expression at a post-transcriptional level. Serum ex-miRNAs are increased in diseases that induce tissue damage and represent tissue-specific cytotoxicity markers [2]. The release of miR-21, ubiquitous and abundant miRNA [37], was found to be related to necrosis [3].

## 3. Biology of Tumor Necrosis and Extra-Cellular Necrotic Products

**Figure 1:**
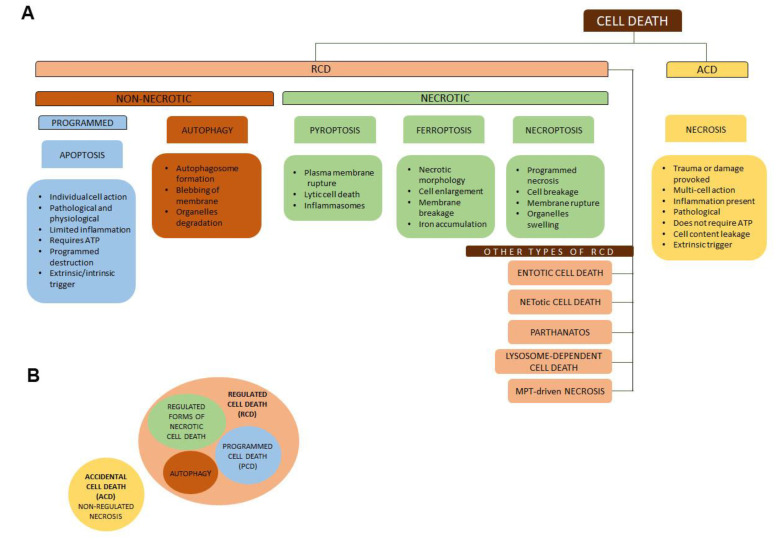
Schematic illustrations of various types of cell death. (**A**) Two broad categories are regulated (RCD) and accidental (ACD) cell death. RCD is the most common type. It includes necrotic and non-necrotic forms of cell death. Non-necrotic cell death includes apoptosis, also called programmed cell death (PCD), and cell death by autophagy. Necrotic regulated cell death includes necroptosis, ferroptosis, and pyroptosis. Other types of RCD are entotic cell death, NETotic cell death, parthanatos, lysosome-dependent cell death, and mitochondrial permeability transition (MPT)-driven necrosis. Accidental cell death usually has the form of a non-regulated necrosis. (**B**) represents the summary of different sorts of cell death.

Cell death was initially categorized into three types: type I cell death (apoptosis), type II cell death (autophagy), and type III cell death (necrosis). However, recent studies have identified additional types of cell death. They are classified based on their biochemical properties, functional potential, and morphology according to the Nomenclature Committee on Cell Death (NCCD). The types of cell deaths described include necroptosis, immunogenic cell death, apoptosis (intrinsic and extrinsic), cellular senescence, pyroptosis, lysosome-dependent cell death, mitochondrial permeability transition (MPT)-driven necrosis, entotic cell death, ferroptosis, parthanatos, NETotic cell death, lysosome-dependent cell death, mitotic catastrophe, and autophagy-dependent cell death [38] (Figure 1).

Besides their differences, apoptosis, autophagy, and necrosis share some common characteristics depending on the stimulatory onset and the signaling pathway that follows afterward. When comparing necrosis and apoptosis, the main difference is the cause of cell death. Apoptosis is a programmed process of cellular death that is a result of genetically-induced cell self-destruction with members of the Bcl-2 family and the caspases 3, 7, 8, 9, and 10 as key regulators. Apoptosis is a part of the natural and preplanned cellular mechanisms that allows the system to maintain the balance of multiplication, and to thus maintain the smooth functioning of the body. Meaning, if cells do not undergo their programmed death, it can lead to cancer formation and the accumulation of redundant cells. Apoptotic cell remnants are recognized and destroyed by the immune system.

Necrosis is also a process of cellular death; however, it happens when the cell is exposed to environmental conditions that are not physiological. The result is the destruction of the inner cellular components causing swift cellular and tissue destruction, leading to inflammation that ends with cell death. Necrosis is a pathological process that can be caused by anything, from a change in oxygen level, pathogens, toxins, and temperature leading to damage of the cell membrane. Necrosis is a random unregulated event that does not require energy on a biochemical level, while apoptosis does require energy as it is an active process [39]. 

Necroptosis is a type of necrosis that is triggered by innate immune activity and results in the rupture of dead cells with leakage of intracellular elements. Necroptosis is a programmed form of necrosis that comes from environmental factors. Necroptosis is a hybrid of necrosis and apoptosis: it is a regulated necrosis mediated by cellular death receptors. Unlike necrosis, necroptosis is a highly regulated programmed cell death, which unlike apoptosis, does not involve caspase activation. It shares some similarities with necrosis, such as the loss of ATP, swelling of the cell, generation of ROS, and release of lysosomal enzymes. Necroptosis has been implicated in the pathology of many diseases, such as acute tissue damage, ischemia-reperfusion injury, stroke, and myocardial infarction [40]. In apoptosis, cytokines release is either not present or significantly decreased. However, in necroptosis, the release of inflammatory cytokines is one of the hallmarks. It is strongly associated with robust inflammation that induces immune activation. Necroptosis is specific to vertebrates and may have developed as a supportive mechanism against pathogens. Pathogens have developed a system of survival within the attacked cell by utilizing virus-encoded inhibitors of caspase activity that can block caspase (and apoptosis), therefore, necroptosis assures cell death. It is a reliable mechanism during viral infection which leads to anti-viral inflammation [41].

### 3.1. Stages in Necrosis

Necrosis is considered an irreversible injury due to membrane damage. It starts with a persistent cell injury as a result of a pathological process where, after a certain point, the injury is irreversible. It is caused by the environmental conditions of the exposed cell, which are not physiological to the cell. Stages of necrosis start with swelling of the cell. It continues to the chromatin digestion and membrane disruption. The inner content of the cell, vacuoles, and organelles break down and the cell is decayed. This leads to leakage of the intracellular content, which provokes the inflammation and the immune response. Necrosis leads to a completely irreversible state of the cell since the final stage is enzymatic degradation of the cell. 

### 3.2. Relationships with Hypoxia—Metabolic Reprogramming (Warburg Effect)

Cancer cells are ravenous due to their steady inappropriate growth. They tend to shape their environment to ensure their proliferation, despite poor blood and oxygen supply, and, therefore, require metabolic reprogramming. In addition to their survival and uninterrupted proliferation, the metabolic reprogramming of malignant cells often facilitates tissue invasion, immune escape, and resistance to therapy. At the heart of this metabolic reprogramming is the Warburg effect, which is a tendency to switch off oxidative phosphorylation and to activate non-mitochondrial glycolysis. Incoming glucose is converted to pyruvate, but instead of entering the citric acid cycle, most of the pyruvate is converted to lactate. One advantage of non-mitochondrial glycolysis for the malignant cells is to allow the production of ATP in the absence of oxygen, albeit with a very low yield compared to oxidative phosphorylation (overall production of 2 ATPs for one glucose molecule instead of 32 ATPs). This explains why the malignant cells are often very hungry for glucose. Another consequence of the Warburg effect is to favor a flux of substrates towards biosynthetic pathways enabling the synthesis of the nucleic acids, proteins, and lipids that are necessary for tumor cell survival and proliferation. Another consequence is the increase of lactate concentration in the tumor microenvironment (TME) because lactate can freely diffuse across biological membranes. Lowering the pH in the TME seems to favor tumor invasion [42].

### 3.3. General Features of Extracellular Products Released by Necrotic Cells: Damage-Associated Molecular Patterns (DAMPs)

Damage-associated molecular patterns (DAMPs), also called alarmins or danger signals, are endogenous molecules released from the cells exposed to different stressors, especially following injury or cell death. After ligation to their specific receptors, they act as sensors, inducers, or mediators of stress or immune response. DAMP receptors include advanced glycosylation end product-specific receptor (RAGE/AGER), Toll-like receptors (TLRs), NOD1-like receptors (NLRs), RIG-I-like receptors (RLRs), AIM2-like receptors (ALRs), transmembrane C-type lectin receptors, P2 × 7, P2Y2, CD91, CD14, CD36, and FPR1 [43] (Table 1). 

DAMPs usually have normal functions inside the cell of origin, however, after release, their function is usually altered. Production of intracellular DAMPs may increase genomic instability [44,45], epigenetic, and telomere modifications [46,47], while extracellular DAMPs induce inflammation [31,48,49] that can contribute on the long term to cancer development. DAMPs may also be involved in the metabolic re-programming towards non-mitochondrial glycolysis, which is often associated to inflammatory processes. For example, this induces metabolic switch, which contributes to the release of HMGB1 in sepsis [50]. Reciprocally, a recent study demonstrated that extra-cellular HMGB1/RAGE promote anaerobic glycolysis of fibroblasts that is required for their activation by breast cancer cells, leading to breast cancer cell metastasis [51].

#### 3.3.1. HMGB1 and RAGE Receptor

One of the most studied DAMPs is HMGB1, which belongs to a group of non-histone nuclear proteins. HMGB1 is an evolutionary highly-conserved protein and appears to be essential for mammalian organisms: HMGB1 knock-out mice live very shortly [52]. It is a nuclear protein that acts as a chromatin-binding factor and DNA chaperone, and is responsible for numerous DNA-associated processes (replication, transcription, recombination, and repair) [53]. HMGB1 has a dual function, which depends on whether it is inside or outside the cell. Loss of intracellular HMGB1 increases DNA damage, genomic instability, cell death, and nuclear DAMP release. Contrarily, extracellular HMGB1 functions as a regulator of inflammation, immunity, metabolism, migration, and autophagy [54]. The basic mechanism for HMGB1 release is oxidative stress and it has been shown that several antioxidants may prevent or reduce its secretion [55,56,57]. However, HMGB1 release may be mediated by several other processes: post-transcriptional modifications (acetylation, ADP-ribosylation, methylation, phosphorylation, glycosylation and oxidation) [54,58,59], nuclear export receptor (chromosome-region maintenance (CRM1)) [60], pyroptosis [61], apoptosis [62], necrosis [31], and autophagy [63]. Receptors that bind HMGB1 are RAGE, TLR2, and TLR4, which in turn activate the MAPKs, NF-κB, and PI3K/AKT signaling pathways [54]. Generally, intracellular HMGB1 acts as a tumor suppressor and may enhance other tumor suppressors’ activity [64]. Extracellular HMGB1 acts as a tumor promoter by accelerating cancer development. By binding to its receptors (RAGE and TLRs), it can enhance multiple aspects of the malignant phenotype. Direct effects can be observed in vitro, for example, the enhancement of tumor sphere formation. The effects observed in vivo are remarkably diverse—metabolic changes, epithelial to mesenchymal transition (EMT), stimulation of autophagy, enhancement of immune suppression, local invasion, angiogenesis, metastasis, radio-resistance, and chemoresistance. These protumoral effects are probably due a direct impact of HMGB1 on malignant cells and to indirect mechanisms involved in inflammatory processes [65,66,67,68,69,70,71,72,73,74,75,76,77]. 

The receptor for advanced glycation products (RAGE) is a pattern recognition receptor (PRR) involved in the recognition of endogenous molecules released from tissue damage. It is a single transmembrane receptor that is a member of the immunoglobulin superfamily [78]. Ligands for RAGE include advanced glycation end products (AGE), members of the S100 family, extracellular HMGB1, amyloid β peptide and amyloid fibrils, β2 integrin Mac-1, glycosaminoglycans and lysophosphaditic acid [79,80,81]. Following ligand binding to RAGE, adaptor proteins (TIRAP, MyD88, diaphanous-1) associate with the RAGE cytoplasmic domain, resulting in signal transduction. The main signaling pathways activated by RAGE include Rho GTPases (cell migration), NF-κB (inflammation), and mitogen-activated protein kinases (MAPK, proliferation) [82]. A soluble form of RAGE (sRAGE) is a naturally occurring competitive inhibitor of RAGE. It originates from the receptor’s ectodomain shedding (cleaved RAGE) or splice variant (endogenous secretory RAGE) [83]. 

#### 3.3.2. Other Proteins Released by Necrotic Cells

##### Histones

Following infection, sterile inflammation, or cell death (apoptosis, necrosis, NETosis), histones as well as nucleosomes are released from the cells. Extra-cellular histones can bind TLRs of neighboring cells (2, 4, and 9) [84]. Histone binding may activate several signaling pathways including MAPKs, NF-κB, and MyD88 [85].

##### S100

The S100 protein family consists of 24 low molecular weight proteins (9–13 kDa) which form homo-, hetero- and, oligomers. Intracellular S100 proteins are involved in many important cellular processes: Ca^2+^ homeostasis, energy metabolism, apoptosis, cell differentiation and proliferation, inflammation, migration and cytoskeletal interactions, protein phosphorylation, and degradation [86]. Extracellular S100 have been detected in the extracellular space and body fluids where they are associated with different diseases. In the TME, S100 proteins contribute to the formation of the pre-metastatic niche, neutrophil extracellular traps (NETs), and activation of the immune response [53].

##### Heat Shock Proteins (HSPs)

Heat shock proteins (HSPs) are conserved ubiquitously-expressed proteins that are overexpressed in the conditions of cellular stress (hyperthermia, hypoxia, changes in pH, toxins, etc.). HSPs were named according to their molecular mass and include HSP27, HSP40, HSP60, HSP70, HSP90, and large HSPs (HSP110 and glucose-regulated protein 170, GRP170) [87]. They are molecular chaperones, which means their main function is to ensure proper protein folding and activation of signaling proteins. If HSPs are dysfunctional, misfolded proteins form aggregates, leading to cell death. 

##### Annexin A1/FPR1

Formyl peptide receptor 1 (FPR1) is a PRR that recognizes N-formylated peptides from bacteria. However, it can also serve as a receptor for DAMPs, such as annexin A1. 

#### 3.3.3. Lipids and Carbohydrates Released by Necrotic Cells

Serum amyloid A protein (SAA) is a lipoprotein involved in cholesterol transport and the production of pro-inflammatory cytokines [88]. SAA is a known ligand for TLR2 and TLR4. Hyaluronic acid (HA) is a polysaccharide that is a major component of the extracellular matrix and also an endogenous ligand for TLR2, TLR4, and, NLRP3.

#### 3.3.4. Metabolite-Related DAMPs

##### ATP

Adenosine 5′-triphosphate (ATP) is a nucleotide present in all living cells, and its main role is in energy metabolism. However, in addition to its intracellular role, extracellular ATP is involved in other important biological processes, such as neurotransmission, inflammation, bone and liver glycogen metabolism, cardiac function, and vasodilatation [89].

##### Uric Acid

Extracellular uric acid originates from intracellular stores of uric acid and enzymatic degradation of purine nucleotides. 

#### 3.3.5. Nucleic Acids Released by Necrotic Cells

Genomic DNA in its B-form is capable of immune system activation when present in the cytosol [90]. Other forms of DNA that may induce the immune system are mitochondrial DNA [91] and single-stranded DNA (AT-rich stem-loop regions) [92]. The DNA-binding receptor is TLR9. Kariko et al. showed that mRNA is an endogenous ligand for TLR3 and that RNA released from necrotic cells may induce an inflammatory response [93]. Additionally, UV irradiation-induced the release of RNA from keratinocytes, which activates TLR3 resulting in the production of inflammatory cytokines [94].

### 3.4. Necrotic Products and Tumor Microenvironment

**Figure 2:**
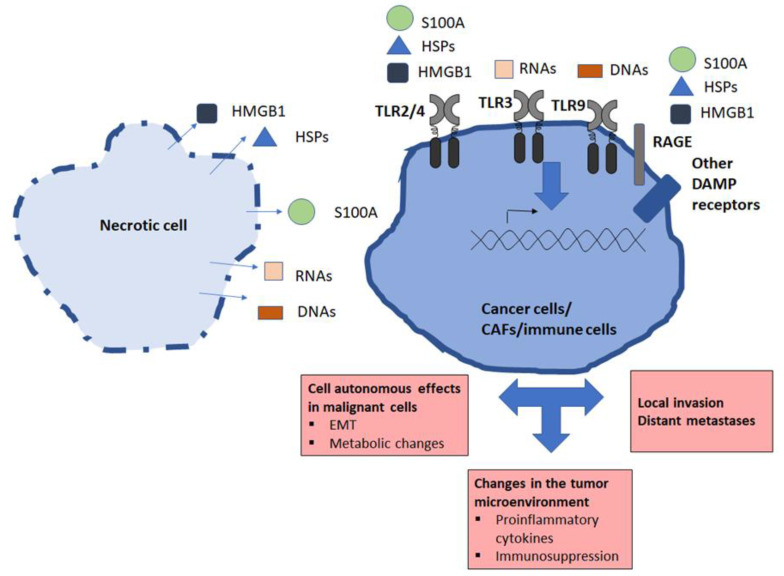
Impact of biomolecules released by necrotic cells on malignant and stromal live cells in the tumor microenvironment (TME). Necrotic cells release DAMPs (HMGB1, HSPs, S100A, RNAs, and DNAs) into the TME. Malignant cells, cancer-associated fibroblasts (CAFs), and various types of infiltrating immune cells have pattern recognition receptors (PRRs). When binding these PRRs, damage-associated molecular patterns (DAMPs) can induce substantial changes in gene expression, resulting in inflammation, epithelial to mesenchymal transition (EMT), immunosuppression, local invasion, and metastases.

In recent years, the critical role of the tumor microenvironment (TME) in cancer initiation and progression has been recognized. Released DAMPs can crucially impact the TME by enhancing vascular stroma formation and angiogenesis, and/or by modifying the immune response (Figure 2). Secretion of HMGB1 from cancer-associated fibroblasts (CAFs) promotes metastatic potential of non-small cell lung cancer cells [95]. HMGB1 signaling between esophageal adenocarcinoma cells and macrophages in the vicinity forms an inflammatory TME, which aids cancer progression [96]. Furthermore, exosomal HMGB1 promotes cancer cell survival, protects cells from doxorubicin cytotoxicity [97] and increases angiogenesis [98]. Secreted HSP90 plays an important role in cancer cell invasion through both binding to the surface receptors, such as CD91, and interacting with matrix metalloprotease 2 on the cellular surface. HSP90 mediates invasiveness, EMT, and modulation of the immune system response [99,100,101]. Ignacio et al. revealed that SAA can predispose inflammatory TME in triple-negative breast cancer [102]. Cancer and CAFs produce SAA, which in the TME can contribute to tumor initiation, progression, metastasis, and immune suppression [103,104,105]. DNA released from neutrophils activates pancreatic stellate cells that form compact, fibrous stroma that can promote and facilitate tumor proliferation [106]. In epithelial ovarian cancer, mtDNA in the TME induces NET formation and suppressive neutrophils, therefore, facilitating metastasis and obstructing the anti-tumor immunity [107]. Extracellular DNA in the TME promotes colorectal tumor cell survival after chemotherapy through induction of autophagy via TLR-9 signaling [108]. Furthermore, many recent studies show DAMPs are released from cancer cells in extracellular vesicles, hence, enabling their dissemination to distant organs (reviewed in [109]). Nabet et al. revealed that breast cancer stromal fibroblasts shed exosomes containing RNA that, in its protein-free/unshielded form, induces RIG-I signaling in breast cancer cells, leading to tumor growth, metastasis, and therapy resistance [110]. 

## 4. Direct Impact of Necrotic Products on Malignant Cells—Role of TLR Ligands

### 4.1. Role of TLR Ligands, Especially TLR3 Ligands

TLR ligands are important for cancer development and progression through their ability to stimulate chronic inflammation and activate signaling pathways, leading to the upregulation of molecules involved in cell proliferation, invasion, and metastasis, or decreased apoptosis [111]. TLR3 ligands are dsRNAs often released by necrotic cancer cells. Liu et al. revealed the mechanism by which TLR3 is involved in pre-metastatic niche formation. Primary tumor releases tumor-derived exosomes, which contain small nuclear RNAs that can activate TLR3 in alveolar cells to produce chemokines and induce neutrophil infiltration [112]. Another study showed a direct link between TLR3 activation by extracellular HSP27 and angiogenesis [113]. We have previously shown that TLR3 activation in head and neck cancer cells can induce metabolic reprogramming and the Warburg effect. TLR3 stimulation induced cancer growth under low serum conditions and metabolic switch from oxidative phosphorylation to extra-mitochondrial glycolysis in a HIF-1α dependent mechanism [114]. In another study, we have demonstrated that, besides metabolic changes, TLR3 activation increases cancer cell migration, ROS production, and decreases anti-oxidative response [115]. All this evidence indicates that TLR3 activation in a tumor and its microenvironment by endogenous ligands can induce tumor survival and progression. A recent study showed that TLR3 overexpression in prostate cancer cells induces cancer invasion while its activation triggers apoptosis, confirming once again the double-edged sword nature of TLR3 [116]. Tavora et al. recently revealed that endothelial cells have a direct instructive role in driving metastatic dissemination: tumor-derived dsRNAs induce TLR3 and SLIT2, leading to intravasation [117]. Bugge et al. established that non-metastatic, healthy intestinal epithelial cells do not express TLR3, while metastatic cells do and that TLR3 promotes their invasiveness [118].

### 4.2. HMGB1 and RAGE

Overexpression of HMGB1 in cancer tissue and increased HMGB1 serum levels have been documented for almost all solid tumors: colon, gastric, lung, breast, ovarian, pancreatic, and prostate [54]. Hoste et al. demonstrated that TLR5 and HMGB1 are crucial in chronic inflammation, tissue damage, and skin cancer induction [119]. During hypoxia, HMGB1 may translocate to cytoplasm where it binds mtDNA and activates TLR9, resulting in hepatocellular carcinoma growth [120]. HMGB1 and its receptor TLR2 have a crucial role in mammary cancer stem cell self-renewal, tumorigenesis, and metastatic ability [121,122]. Irradiated colorectal tumor cells stimulate non-irradiated tumor cell proliferation when co-cultured through HMGB1 [123], while in bladder cancer, HMGB1 was connected with radioresistance, in vitro and in vivo, through the upregulation of autophagy [124]. Melanoma tumor cells release HMGB1 in response to hypoxia, which promotes M2-like tumor-associated macrophage accumulation and IL-10 production, leading to an increased tumor growth and metastasis [71]. Zhu et al. revealed that the redox state of HMGB1 is crucial in colorectal carcinoma angiogenesis. The all-thiol variant of HMGB1 interacting with RAGE was important for endothelial cell migration while disulfide HMGB1 binding to TLR4 was essential for VEGF up-regulation [125]. Chen et al. demonstrated how TLR2 and TLR4 have completely different roles during interactions with HMGB1: HMGB1 released from dying cells after radiotherapy contributed to stemness maintenance via interaction with TLR2, however, TLR4 antagonized this process [126]. Recent studies revealed how HMGB1 is involved in immunosuppression through galectin-9 induction [127] and chemotherapy resistance [128].

RAGE has been connected with tumorigenesis, cancer growth, and metastasis, probably through cancer-induced carbonyl stress (increased production of AGE) and hypoxia. The overexpression of RAGE and its ligands was detected in different cancer tissues [78]. AGEs derived from glucose may promote the invasion and metastasis of colorectal cancer through the RAGE/ERK/SP1/MMP2 axis [129]. RAGE is important for breast cancer cell invasion and metastasis in vitro and in vivo [130]. In melanoma cells, the interaction of extracellular S100A4 with RAGE induced pre-metastatic events, such as increased migration, invasion, and reduced adhesion [131]. RAGE was also associated with endometrial [132], prostate [133], and liver cancer progression [134]. All these findings point out the extreme importance of HMGB1 and RAGE in cancer development and dissemination, especially as many studies show HMGB1 may arise from cancer chemo- and radiotherapy-induced necrotic cell death.

### 4.3. Other Product and Receptors 

TLR4 signaling is activated by lipopolysaccharide (LPS) originating from gram-negative bacteria. However, endogenous fatty acids can also activate TLR4, which makes this receptor a molecular link between nutrition, lipids, and inflammation [135,136]. Additionally, as pro-inflammatory cytokines production is dysregulated in obese adipose tissue, obesity may be observed and studied as an inflammatory disease caused by fatty acids serving as DAMPs molecules [137]. Iannucci et al. also showed recently that TLR4 can mediate inflammation by extracellular IFI16, a novel DAMP and TLR4 ligand, which is released from chronically inflamed tissue [138]. Several recent studies demonstrated the role of TLR4 in the creation of an immunosuppressive environment: by stimulating immunosuppressive myeloid cells [139], by small extracellular vesicles released from malignant cells [140], and by HMGB1 stimulation of TLR4 resulting in the production of immunosuppressive protein galectin-9 [127]. 

There are not many studies about SAA and HA serving as DAMPs in cancer regulation. It was shown that TLR4 induction by SAA3 leads to facilitated metastasis through the NF-κB signaling pathway [141]. Small fragments of the extracellular matrix component HA (sHA) also enhance the motility of cancer cells through the TLR4 signaling pathway in melanoma [142] and papillary thyroid carcinoma [143]. HA binds to TLR4, resulting in proliferation and apoptosis inhibition in colon cancer cells [144].

The S100 family of proteins have been implicated in the promotion of growth and dissemination of many different types of cancer: hepatocellular [145], colon [146], hypopharyngeal [147], prostate [148], endometrial [149], melanoma [131], breast [150], glioma [151], thyroid [152], renal [153], lung [154], and pancreas [155]. Zhuang et al. recently demonstrated that overexpression of S100A2, S100A6, S100A10, S100A11, S100A14, and S100A16 was associated with higher T-stage, advanced histologic grade, worse prognosis, and impaired immune response in pancreatic cancer [156]. S100 proteins seem to be important in the interplay between cancer cells and immune cells in the TME. Fang et al. demonstrated that S100A9 expression in monocytes stimulated the aggressiveness of co-cultured oral cancer cells [157]. Tumor-infiltrating monocytes/macrophages in the TME play an important role in promoting tumor invasion and migration by upregulating S100A8 and S100A9 expression in cancer cells [158], while a high number of S100A9-positive inflammatory cells in cancer stroma is associated with poor outcomes in prostate cancer patients [159]. RAGE and S100A7 also modulate TME by recruiting tumor-associated macrophages (TAMs) in breast cancer [160]. Jo et al. also recently observed that co-culture of normal cells with breast cancer cells induced EMT and increased proliferation, migration, and sphere formation, which was linked to S100A8/9 overexpression [161]. Moreover, Shen et al. demonstrated that transcription factor SOX9 regulates S100P expression, resulting in metastasis and invasion of colon carcinoma [162]. S100A4 stimulation was also connected with metabolic reprogramming in melanoma [163]. 

HSPs are often overexpressed in tumors due to the stressful conditions in the TME, leading to hypoxia, acidity, and deprivation of nutrients. Increased HSPs levels may also cause impaired apoptotic response and promote tumor growth by stabilizing proteins involved in cancer survival [164,165] as well as promote radioresistance [166] or EMT [167,168]. HSPs are also the key target proteins in novel cancer therapies (recently reviewed in [169]) since they may promote chemoresistance [170,171]. Moreover, HSP90α converts monocytes to immunosuppressive myeloid cells in melanoma through TLR4 signaling [139]. A recent study demonstrated that plasma HSP90α level can be used as a prognostic biomarker for hepatocellular carcinoma [172]. HSP90 inhibition might be a novel strategy for advanced papillary renal cell carcinoma [173], metastatic triple-negative breast cancer [174], pancreatic carcinoma [175], prostate cancer [176], and glioma [177] treatment. HSP90 inhibition also improves the survival of patients with gastrointestinal stromal tumors [178] and overcomes resistance to molecular targeted therapy in glioma [179]. HSP27 associates with EMT and stemness in several different types of cancer [180,181,182,183]. The potential of HSP27 inhibition as a target for cancer therapy has also recently been reviewed [184]. 

An interesting novel study by Katakam et al. revealed that DAMPs released by necrotic tumor cells promote the growth of spheroids but not 2D cultures of Ewing sarcoma. Stimulation by DAMPs of cells grown in 3D resulted in an increased expression of genes associated with cholesterol synthesis and in enhanced cellular cholesterol load. On the other hand, activation of the stimulator of interferon genes (STING) by its natural ligand cGAMP inhibited cell growth and reduced the cellular cholesterol load. This reveals a link between the innate immune response driven by STING and cholesterol homeostasis, which may have important implications for tumor growth. [185].

## 5. Impacts of Necrotic Products on the Other Components of the Tumor Microenvironment

### 5.1. Recruitment and Action of Immune Cells 

DAMPs can promote cancer growth and progression by acting upon the immune system via enhanced inflammation and through immunosuppression. DAMPs released from necrotic cells act as chemo-attractants and initiate the immune response. Phagocytosis represents one of the preventive responses against released necrotic debris. Macrophages recruited to pre-necrotic zones are capable of only a limited number of cycles of clearing debris after which they downregulate their phagocytic machinery [186]. The hypoxic microenvironment also initiates sequential changes from anti-tumor M1 to pro-tumor M2 macrophage phenotype. TAMs increase in number as the tumor grows. In some tumors, TAMs contribute to a significant proportion of the tumor mass, and are associated with disease progression and poor prognosis [186,187]. 

Eosinophil infiltration of tumors is observed from the earliest palpable stages, with significant accumulations only in the necrotic and capsule zones of solid tumors [187]. DAMPs present in the necrotic zone induce eosinophil recruitment, their degranulation (the release of toxic cationic granule proteins), and oxidative burst (the release of ROS). Eosinophils are thus capable of inducing an oxidative environment and have a role in the inactivation and clearance of necrotic debris [188,189]. 

### 5.2. Deleterious Effects of Neutrophils 

Immune responses induced by HMGB1 strongly depend on its oxidation status. Necrotic cells release a fully active, reduced form of HMGB1. That form, by binding on its receptors, induces the release of proinflammatory cytokines, creating an inflammatory microenvironment. HMGB1 also promotes the recruitment of inflammatory cells, preferentially neutrophils [190]. Neutrophil infiltration is observed in pre-clinical and clinical cancer models and contributes to tumor cell proliferation and metastasis. The high levels of neutrophil infiltrate, due to the release of high levels of ROS and cytotoxic compounds, promote tumor necrosis, sustain chronic inflammation, and negatively correlate with prognosis and survival [190,191]. Simultaneously with the infiltration of neutrophils, cytokines released from tumor cells cause pathologically-enhanced hematopoiesis, skewed from lymphocytic to granulocytic, that produces more neutrophils [189]. A high neutrophil-to-lymphocyte ratio in the peripheral blood is shown to be associated with poor outcomes in many solid tumors [191]. Overproduction in bone marrow gives rise to immature neutrophils that lack cytotoxic granules and are immunosuppressive. New cohorts of immature cells represent a heterogeneous population of immature myeloid-derived suppressor cells (MDSCs) that promote tumor growth and suppress other effector cells (cytotoxic T lymphocytes and NK cells) [190].

### 5.3. Immunosuppressive Effects of Necrotic Products

Extracellular adenosine is a potent immunosuppressive metabolite. Its level is normally low, however, in response to hypoxic stimulation and inflammation, adenosine level can be induced over a hundred fold [192]. Adenosine, through its receptor A2aR, affects T cells, Treg cells [193], NK cells, and myeloid-derived suppressor cells, leading to an immunosuppressive effect. Blockade of A2aR enhanced NK cell maturation and cytotoxic function, reduced metastasis [194], and increased the number of tumor-infiltrating cytotoxic lymphocytes and decreased Treg cells [195]. ATP was also shown to be involved in immunosuppression: MDSCs from tumor-bearing mice express P2X7 receptor, which promotes the release of immunosuppressive cytokines after triggering with ATP [196]. Baghdadi et al. showed that DAMPs released from chemotherapy-damaged tumor cells upregulate T cell immunoglobulin and mucin domain-containing molecule-4 (TIM-4) on tumor-associated myeloid cells, leading to the repression of tumor-specific immunity [197]. HMGB1 may suppress the antitumor immune response by interacting with TIM-3 [198] and through the promotion of Treg cells survival while limiting the functional activity of conventional T cells [199]. HMGB1 may also act through RAGE and affect pDCs to induce a tolerogenic response during cervical/vulvar carcinogenesis [200]. A recent study showed that HMGB1 derived from hepatocellular carcinoma triggers M2 macrophage polarization through TLR2 and autophagy [201]. Exosome-derived HMGB1 may activate B cells and promote TIM-1+Breg cell expansion via the TLR2/4 and MAPK signaling pathways, therefore, generating an immunosuppressive milieu [202]. HMGB1 also mediates immune suppression through the upregulation of PD-L1 [203]. 

S100A8/A9 induces the accumulation of MDSCs and is secreted by MDSCs and tumor cells, which forms a positive autocrine feedback loop within the inflammatory tumor environment [204]. Similarly, Cheng et al. demonstrated that S100A9, whose expression is regulated by STAT3, is crucial for the inhibition of DC differentiation and the stimulation of the accumulation of MDSCs in cancer [205]. Moreover, overexpression of S100A6, S100A10, S100A11, S100A14, and S100A16 may impair the infiltration and cytolytic activity of cytotoxic lymphocytes in pancreatic cancer [156]. HP70 and HSP90α both induce the immunosuppressive effect of MDSCs [139,206].

Recent findings demonstrated that DAMPs are able to induce NET formation [207]. A study by Munir et al. provided insight into a novel mechanism by which CAFs stimulate t-NETosis at local and systemic levels via the production of amyloid β that serves as DAMP [208]. Several studies have shown that different kinds of traumas induce DAMPs in patients’ plasma resulting in immune suppression [209,210].

Contrary to all this, DAMPs also act as a double-edged sword by inhibiting cancer progression via immunogenic cell death (ICD). Important effectors of ICD are calreticulin, ATP, and HMGB1 [211,212,213]. This has recently been reviewed by [214].

## 6. Conclusions

DAMPs are produced and released in the TME by cells dying from spontaneous necrosis mainly linked to nutrients/oxygen deprivation. They can also be released by cells dying from the action of therapeutic agents during chemotherapy or radiotherapy. DAMPs can bind different receptors, including TLRs, either on cancer cells, immune cells or other cells in the TME, such as CAFs or other stromal cells. Since TLR agonists, and especially TLR3 agonists because of their ability to induce apoptosis, are already being used in various clinical studies, either in form of anticancer drugs or as immunoadjuvants, their potential detrimental role must again be emphasized. Further studies are needed in order to reveal which signaling pathways induce cell death and what are those pathways or conditions that promote tumorigenesis before the introduction of TLR ligands into clinical practice.

## Figures and Tables

**Table 1:** List of damage-associated molecular patterns (DAMPs) and their receptors.

Type of DAMP	DAMPs	Receptors
Proteins	HMGB1	TLR2, TLR4, RAGE
	Histone	TLR2, TLR4
	S100	TLR2, TLR4, RAGE
	HSPs	TLR2, TLR4, CD91, RAGE
	Annexin A1	FPR1
	Versican	TLR2, TLR6, CD14
	Fibronectin (EDA domain)	TLR4
	Fibrinogen	TLR4
	Tenascin C	TLR4
	F-actin	DNGR-1
	Cyclophilin A	CD147
	Aβ	TLR2, NLRP1, NLRP3, CD36, RAGE
	IL1α	IL-1R
	IL33	ST2
	Formyl peptide	FPR1
	Calreticulin	CD91
	Defensins	TLR4
	Cathelicidin (LL37)	P2X7, FPR2
	Granulysin	TLR4
Lipids and carbohydrates	LMW hyaluronan	TLR2, TLR4, NLRP3
	SAA	TLR2, TLR4
	Heparan sulfate	TLR4
Metabolite-related DAMPs	ATP	P2X7, P2Y2
	Uric acid	NLRP3, P2X7
Nucleic acids	DNA	TLR9, AIM2
	RNA	TLR3, TLR7/8, RIG-I, MDA5
	mtDNA	TLR9

## Data Availability

Not applicable.
